# Changes in the epidemiology and clinical characteristics of viral gastroenteritis among hospitalized children in the Mainland of China: a retrospective study from 2016 to 2020

**DOI:** 10.1186/s12887-024-04776-1

**Published:** 2024-05-04

**Authors:** Fei Li, Lingyun Guo, Qi Li, Hui Xu, Yiliang Fu, Luci Huang, Guoshuang Feng, Gang Liu, Xiangpeng Chen, Zhengde Xie

**Affiliations:** 1 Beijing Key Laboratory of Pediatric Respiratory Infection Diseases, Key Laboratory of Major Diseases in Children, Ministry of Education, National Clinical Research Center for Respiratory Diseases, Research Unit of Critical Infection in Children, Chinese Academy of Medical Sciences, 2019RU016, Laboratory of Infection and Virology, Beijing Pediatric Research Institute, Beijing Children’s Hospital, Capital Medical University, National Center for Children’s Health, Beijing, China; 2 Department of Infectious Disease, National Center for Children’s Health, Key Laboratory of Major Diseases in Children, Ministry of Education, Beijing Children’s Hospital, Capital Medical University, Beijing, China; 3 Big Data and Engineering Research Center, Beijing Children’s Hospital, Capital Medical University, National Center for Children’s Health, Beijing, China

**Keywords:** Epidemiology, Viral gastroenteritis, Children, Co-infection

## Abstract

**Background:**

Acute gastroenteritis (AGE) causes significant morbidity in children worldwide; however, the disease burden of children hospitalized with viral gastroenteritis in China has been rarely described. Through this study, we analyzed the data of hospitalized children with viral gastroenteritis to explore the changes in the epidemiology and clinical characteristics of viral gastroenteritis in the mainland of China.

**Methods:**

Data were extracted from Futang Children's Medical Development Research Center (FRCPD), between 2016 and 2020, across 27 hospitals in 7 regions. The demographics, geographic distribution, pathogenic examination results, complications, hospital admission date, length of hospital stays, hospitalization charges and outcomes were collected and analyzed.

**Results:**

Viral etiological agents included rotavirus (RV), adenovirus (ADV), norovirus (NV) and coxsackievirus (CV) that were detected in 25,274 (89.6%), 1,047 (3.7%), 441 (1.5%) and 83 (0.3%) cases. There was a higher prevalence of RV and NV infection among children younger than 3 years of age. RV and NV had the highest detection rates in winter, while ADV in summer. Children with viral gastroenteritis were often accompanied by other diseases, such as myocardial diseases (10.98–31.04%), upper respiratory tract diseases (1.20–20.15%), and seizures (2.41–14.51%). Among those cases, the co-infection rate with other pathogens was 6.28%, with *Mycoplasma pneumoniae* (*M. pneumoniae*), Epstein-Barr virus (EBV), and influenza virus (FLU) being the most common pathogens. The median length of stay was 5 days, and the median cost of hospitalization corresponded to587 US dollars.

**Conclusions:**

This finding suggests that viral gastroenteritis, especially those caused by RV, is a prevalent illness among younger children. Co-infections and the presence of other diseases are common. The seasonality and regional variation of viral etiological agents highlight the need for targeted prevention and control measures. Although viral gastroenteritis rarely leads to death, it also results in a significant economic burden on healthcare systems.

**Supplementary Information:**

The online version contains supplementary material available at 10.1186/s12887-024-04776-1.

## Background

Acute gastroenteritis is a common disease that affects people of all ages, leading to serious complications in young children and the elderly [1–4]. In China, infectious diarrhea (excluding cholera, dysentery and enteric fever) has been classified as a class C infectious disease according to the national notifiable infectious diseases reporting system. Enteric viral pathogens are gradually becoming the leading pathogens of gastroenteritis which is also known as infectious diarrhea, due to the improved the quality of drinking water and the frequent use of antibiotics. Clinical manifestations of viral gastroenteritis include fever, abdominal pain, watery diarrhea, nausea and vomiting. Viral gastroenteritis is usually a self-limiting illness, requiring mainly supportive therapy, which usually resolves within 2–5 days. Viral pathogens include rotavirus (RV), norovirus (NV), astrovirus (AV) and adenovirus (ADV). NV infection affects people of all ages, while RV mainly infects children, particularly those under five years of age [5–8].

The implementation of RV vaccination has reduced RV problems to some extent, but the contribution of RV to pediatric acute gastroenteritis has not been replaced by other pathogens [3, 6, 9]. Vaccination has helped to reduce RV hospitalization, as well as change the epidemiology of RV disease in the United States and Spain [10, 11]. RV vaccine is part of the National Immunization Programs (NIPs) in many countries, but it has not been included in China’s NIPs. The epidemiology and distribution of common pathogens causing infectious gastroenteritis, particularly viruses, are unclear in developing countries including China.

In China, there are few multicenter studies on the clinical epidemiological characteristics and disease burden of viral gastroenteritis in children. By using the hospitals’ electronic medical record management system, the medical data generated during the hospitalization of patients can be summarized into face sheet of discharge medical records (FSMRs). This study aimed to summarize and provide relevant data on the clinical epidemiology and disease burden of viral gastroenteritis in hospitalized children in China.

## Methods

### Study design and participants

In China, Futang Children's Medical Development Research Center (FRCPD) is the first non-profit social service organization established to care for children's lives and health and engage in children’s development research [12], supervised and managed by the Ministry of Civil Affairs of the People’s Republic of China and led by the Children’s Medical Center. The center currently consists of 47 provincial and municipal medical institutions and has established a nationwide children health service network [13]. In Dec 2015, FRCPD began to collect the data of FSMRs from its member hospitals. The National Center for Children's Health (Beijing), Beijing Children’s Hospital, Capital Medical University collected the data of the hospitalized children’s medical records from Jan 1st, 2016 to Dec 31st, 2020 in 27 tertiary children's hospitals under the FRCDP (Supplementary Material 1).

**Fig. 1:**
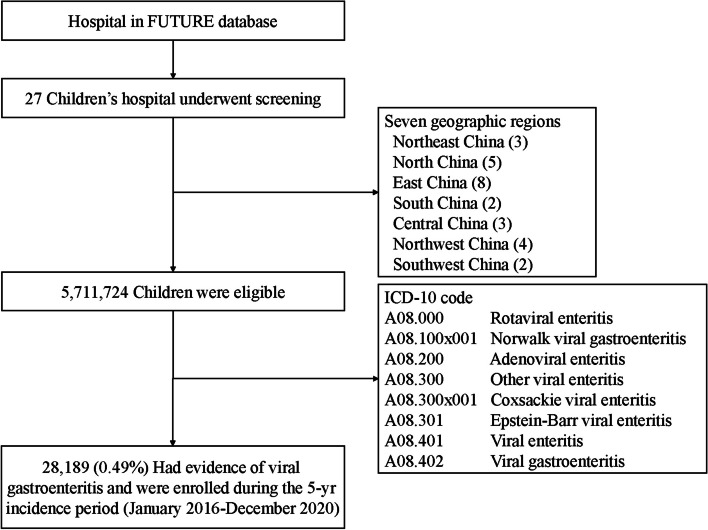
Screening, eligibility, and enrollment of children with viral gastroenteritis. A total of 28,189 children had the clinical and etiological diagnosis that met the inclusion criteria of viral gastroenteritis

We designed this study to collect basic medical information of children hospitalized with viral gastroenteritis in the FUTang Updating medical REcords (FUTURE) database from 2016 to 2020, and extracted relevant information from the system based on the tenth revision of the International Statistical Classification of Diseases and Related Health Problems 10th Revision (ICD-10) code for children diagnosed with viral gastroenteritis (Fig. 1). The demographic information isdisplayed in Table 1. Subgroups were divided according to the sex, age, region, and time of hospitalization status. All hospitals were divided into seven geographic regions. According to different ages, hospitalized children with viral gastroenteritis were divided into six groups, including neonate (≤ 28 days), infant (28 days <  ~  ≤ 1 year old, neonate were excluded), toddler (1 <  ~  ≤ 3 years old), preschooler (3 <  ~  ≤ 6 years old), school–age children (6 <  ~  ≤ 12 years old), adolescence (12 <  ~  < 18 years old).

**Table 1:** Demographics across cohort of patients with confirmation of viral gastroenteritis. Values are positive numbers (rate) unless stated otherwise

Characteristic	Patients (n)	Patients with viral gastroenteritis (n, %)	χ^2^	*P value*
Total	5,711,724	28,189(0.49)		
Sex			16.863	< 0.001
Male	3,532,336	17,099 (0.48)		
Female	2,179,388	11,090 (0.51)		
Age group			23,590.088	< 0.001
≤ 28 days	571,879	733 (0.13)		
28 days < ~ ≤ 1 years old	1,632,150	17,563 (1.08)		
1 < ~ ≤ 3 years old	1,207,254	8803 (0.73)		
3 < ~ ≤ 6 years old	1,099,157	959 (0.09)		
6 < ~ ≤ 12 years old	1,030,936	124 (0.01)		
> 12 years old	170,348	7 (0.00)		
Years			331.482	< 0.001
2016	1,015,850	5102 (0.50)^a^		
2017	1,123,239	6618 (0.59)		
2018	1,205,364	5919 (0.49)^a^		
2019	1,337,314	6129 (0.46)		
2020	1,029,957	4421 (0.43)		
Seasons			11,117.324	< 0.001
Spring	1,362,478	6090(0.45)		
Summer	1,525,852	2731(0.18)		
Autumn	1,412,944	5188(0.37)		
Winter	1,410,450	14,180(1.01)		
Regions			9,236.243	< 0.001
Northeast China	249,244	2023(0.81)		
North China	828,006	3020(0.36)^a^		
East China	1,896,173	6960(0.37)^a^		
South China	422,268	1689(0.40)		
Central China	1,098,999	2671(0.24)		
Northwest China	775,561	8505(1.10)		
Southwest China	441,473	3321(0.75)		
Hospital discharge-dollars				-
Discharge against medical advice		1640 (5.82)		
Other		174 (0.62)		
Discharge with medical advice		26,363 (93.52)		
Transferred to other hospital with medical advice		12 (0.04)		
Death		0(0.00)		
Hospitalization				-
Length of stay- days				
Median		5		
Interquartile range		3–6		
Cost-US dollars				
Median		587		
Interquartile range		541–642		

Letters “a” showed that there was not different between these groups

### Inclusion and exclusion criteria

This retrospective study included only children under the age of 18 who were hospitalized for viral gastroenteritis based on the classification of viral gastroenteritis according to ICD-10 codes, we collected basic medical information from the FUTURE database. Children were diagnosed with viral gastroenteritis according to the clinical and etiological diagnosis [3, 4]. Data of children with unknown sex, age, region or resident condition were excluded.

### Statistical analysis

Continuous variables were presented as mean ± standard deviation (SD) and compared between groups by Student’s t-test when normally distributed. For not normally distributed variables, the data were expressed as median (interquartile range, IQR), and Kruskal–Wallis test as well as Steel–Dwass test (for multiple comparisons) were performed to compare the difference among groups. Categorical variables were expressed as number (%) or proportions and compared between/among groups by χ2 or Fisher’s exact tests, when appropriate. IBM SPSS Statistics 23.0 software (SPSS Inc., USA) was used for data analysis. *P* value < 0.05 was considered statistically significant.

## Results

### Prevalence of viral gastroenteritis in children

**Fig. 2:**
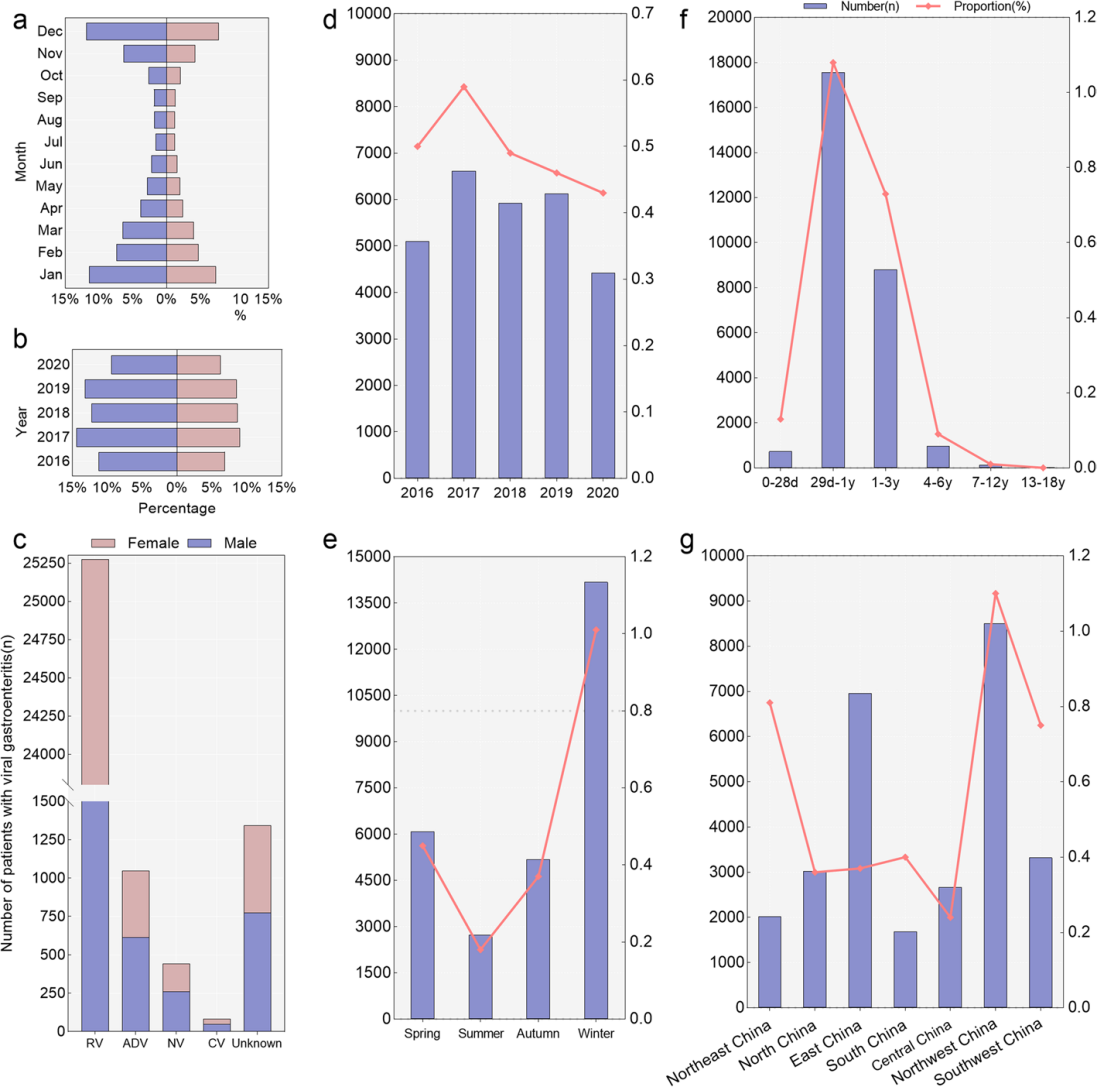
The proportion of children hospitalized for viral gastroenteritis by different gender, years, ages and regions. Panel **a**-**c** show the proportion of different years, months and pathogens according to gender. Panel **d**-**g** show the number and proportion in different years, age groups, seasons and regions of China, the left Y axis (the bars) is the number, while the right Y axis (the line plots) is the proportion of hospitalized children with viral gastroenteritis. The definition of "unknown" is that children with viral gastroenteritis were for an unspecified viral cause

During 2016–2020, a total of 28,189 hospitalized children with viral gastroenteritis were enrolled in the FUTang Updating medical Records (FUTURE) database, which accounted for 0.5% (28,189/5,711,724) of all hospitalized cases, with 60.6% male (17,099) and 39.3% female (11,096) (Table 1). In different month and admission years, the proportions of male were higher compared to female (Fig. 2). The information on sex, age, year, season, regions and outcomes is shown in Table 1.

In different genders, years, regions, and age groups, we evaluated the proportion of viral gastroenteritis hospitalizations to total hospitalization (Table 1, Fig. 2). Females had a higher proportion of children with viral gastroenteritis than males (*P* < *0.001*), with the rates of 0.5% (11,090/2,179,388) and 0.5% (17,099/3,532,336), respectively (Table 1). The rate of viral gastroenteritis in the age group from 29 days to 1 year old was significantly higher than those in other age groups (Table 1) (*P* < *0.001*). The proportion of viral gastroenteritis hospitalizations to total hospitalization also differed with seasonal variability, which was higher in winter and lower in summer (Table 1) (*P* < *0.001*). Northwest China had the highest proportion (1.1%, 8,505/775,561) and Central China had the lowest proportion (0.2%, 2,671/1,098,999) (*P* < *0.001*).

### Viral gastroenteritis characterized by different viral pathogens

**Fig. 3:**
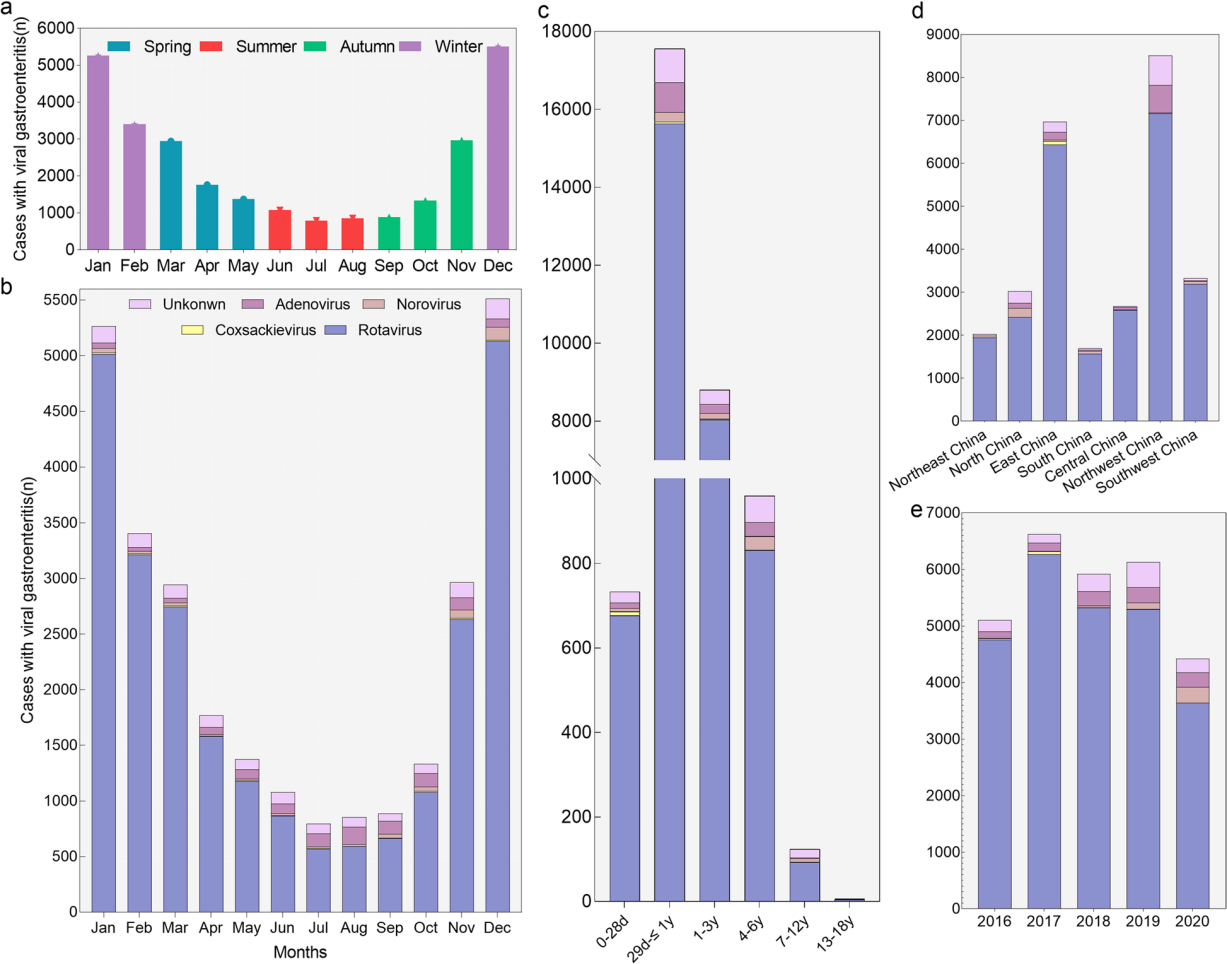
Pathogens detected in children with viral gastroenteritis requiring hospitalization. Panel **a** show the number of hospitalized children with viral gastroenteritis in different seasons. Panel **b** show the pathogens distribution in different months. Seasons were based on months as follows: winter, January through March; spring, April through June; summer, July through September; fall, October through December. Panel **c**-**e** show the pathogens distribution in in different age groups, years and regions of China. The definition of "unknown" is that children with viral gastroenteritis were for an unspecified viral cause

Among those admitted to the hospital with a diagnosis of viral gastroenteritis, 95.2% (26,845/28,189) cases were positive for viral infection (RV, NV, CV and ADV) (Table 2, Fig. 3). RV had the highest positive proportion (89.7%) than the other viruses (*P* < *0.001*). RV, NV, CV and ADV had the highest positive rate in the 28 days <  ~  ≤ 1 year group than other age groups (*P* < *0.001*). RV and NV had the highest detection rate in winter compared with other seasons, while ADV had the highest detection rate in summer (*P* < *0.001*). RV showed high detection rates in Northwest China and East China (28.3% and 25.4%), while NV had a high detection rate in North China, CV in East China and ADV in Northwest China (46.9%, 94.0% and 61.5%) compared to other regions (*P* < *0.001*) (Table 2).

**Table 2:** Positive number(n) and rate (%) of children hospitalized for viral gastroenteritis from 2016 to 2020

Characteristic	CV	RV	NV	ADV	Not clear
Total (*n* = 28,189)	83(0.29)	25,274(89.66)	441(1.56)	1047(3.71)	1344(4.77)
*P value*			<0.001		
Sex					
Male(*n* = 17,099)	51(61.45)	15,395(60.91)	262(59.41)	615(58.74)	776(57.74)
Female(*n* = 11,090)	32(38.55)	9879(30.09)	179(40.59)	432(41.26)	568(42.26)
χ^2^	0.022	6.607	0.292	1.678	5.044
* P value*	0.883	0.010	0.589	0.195	0.025
Age group					
≤ 28 days(*n* = 733)	9(10.84)^a^	677(2.68)^a^	8(1.81)^a^	13(1.24)^a^	26 (1.93)^a,b^
28 days < ~ ≤ 1 years old(*n* = 17,563)	47(56.63)^b^	15,635(61.86)	249(56.46)^a^	764 (72.97)^b,c^	868(64.58)^b^
1 < ~ ≤ 3 years old(*n* = 8803)	26(31.33)^b^	8034(31.79)^a^	142 (32.20)^a^	234(22.35)^a,d,e^	367(27.31)^a^
3 < ~ ≤ 6 years old(*n* = 959)	1 (1.20)^b^	831(3.29)	32(7.26)^b^	33(3.15)^b,c,d,e^	62(4.61)^c^
6 < ~ ≤ 12 years old(*n* = 124)	0(0.00) ^a,b^	93(0.37)^b^	9(2.04)^c^	2(0.19)^a,c,e^	20(1.49)^d^
> 12 years old(*n* = 7)	0(0.00) ^a,b^	4(0.02)^b^	1(0.23)^b,c^	1(0.10)^b,d^	1(0.07)^a,b,c,d^
χ^2^	23.752	83.984	56.675	58.943	53.265
* P value*	< 0.001	< 0.001	< 0.001	< 0.001	< 0.001
Years					
2016(*n* = 5102)	23(27.71)^a^	4754 (18.81)	3(0.68)^a^	120(11.46)^a^	202 (15.03)
2017(*n* = 6618)	45(54.22)^a^	6267(24.80)	12(2.72)^a^	143(13.66)^a^	151(11.24)
2018(*n* = 5919)	11(13.25)	5319(21.05)	33(7.48)	252(24.07)^b^	304(22.62)^a^
2019(*n* = 6129)	3(3.61)^b^	5292(20.94)	117(26.53)	271(25.88)^b^	446(33.18)
2020(*n* = 4421)	1(1.20)^b^	3642(14.41)	276(62.59)	261(24.93)	241 (17.93)^a^
χ^2^	63.841	574.912	829.389	143.848	188.731
* P value*	< 0.001	< 0.001	< 0.001	< 0.001	< 0.001
Seasons					
Spring(*n* = 6090)	19(22.89)	5508(21.79)	56(12.70)	189(18.05)	318(23.66)^a^
Summer(*n* = 2731)	10(12.05)	2028(8.02)	54(12.24)	356(34.00)	283(21.06)
Autumn(*n* = 5188)	18(21.69)	4380(17.33)	152(34.47)	346(33.05)	292(21.73)^a^
Winter(*n* = 14,180)	36(43.37)	13,358(52.85)	179(40.59)	156(14.90)	451(33.56)
χ^2^	1.824	1171.716	90.68	1067.481	278.173
* P value*	0.610	< 0.001	< 0.001	< 0.001	< 0.001
Regions					
Northeast China(*n* = 2023)	0(0.00)^a,b,c,d,e^	1939(7.67)^a^	47(10.66)^a^	0(0.00)	37(2.75)^a^
North China(*n* = 3020)	0(0.001)^d,e^	2418(9.57)	207(46.94)	122(11.65)	273(20.31)^b^
East China(*n* = 6960)	78(93.98)	6428(25.43)^c^	40(9.07)	182(17.38)^a^	232(17.26)
South China(*n* = 1689)	1(1.20)^b,c,e^	1560(6.17)^c^	68(15.42)	25(2.39)^b^	35(2.60)^a^
Central China(*n* = 2671)	4(4.82)^c^	2583(10.22)^a^	4(0.91)^b^	53(5.06)^a,b^	27 (2.01)
Northwest China(*n* = 8505)	0(0.00)^a,d^	7156(28.31)	19(4.31)^b^	644(61.51)	686(51.04)^b^
Southwest China(*n* = 3321)	0(0.00)^a,b,d,e^	3190(12.62)^a^	56(12.70)^a^	21(2.01)	54(4.02)^a^
χ^2^	216.754	1020.363	801.414	590.509	577.404
* P value*	< 0.001	< 0.001	< 0.001	< 0.001	< 0.001
Hospital discharge, n (proportion, %)					
Against medical advice(*n* = 1640)	4(4.82)	1461(5.78)	44(9.98)	52(4.97)	79(5.88)
Other(*n* = 174)	2(2.41)	165(0.65)	0(0.00)	3(0.29)	4(0.30)
With medical advice(*n* = 26,363)	77(92.77)	23,637(93.52)	397(90.02)	992(94.75)	1260(93.75)
Transferred to other hospital(*n* = 12)	0(0.00)	11(0.04)	0(0.00)	0(0.00)	1(0.07)

Letters “a” “b” “c” “d” and “e” showed that there was different between groups in the different groups

*CV* Coxsackievirus, *RV* Rotavirus, *NV* Norovirus, *ADV* Adenovirus

### Viral gastroenteritis combined with other diseases

Children hospitalized with viral gastroenteritis were often presented with one or more coexisting diseases. Among the 25,274 cases of RV-positive children, they had various other diseases. Specifically, 31.04% had myocardial diseases, 14.41% had upper respiratory tract diseases, 11.83% had bronchitis, and 6.92% had bronchopneumonia. Meantime, myocardial diseases and upper respiratory tract diseases are also common in children infected with CV, NV and ADV (Table 3). Among positive cases of CV (23 cases), RV (7845 cases), NV (75 cases), and ADV (115 cases) with myocardial diseases, myocardial injury rates were highest for cases of myocardial impairment, with 100.00% (23 cases), 78.88% (6188 cases), 95.0% (72 cases), and 73.04% (84 cases), respectively. Nervous system diseases such as seizure accounted for 14.51% of children with NV, 6.02% with ADV, 5.60% with RV and 2.41% with CV. Among RV-positive children with seizures, there were 14 cases of febrile seizures (0.99%), 31 cases of benign convulsions with gastroenteritis (2.19%), and 218 cases of benign infantile convulsions (15.41%). Moreover, other diseases were relatively low, such as urogenital diseases, and nutritional diseases (Table 3).

**Table 3:** Positive number(n) and rate (%) of children hospitalized for viral gastroenteritis with different diseases

Diseases	CV (*n* = 83)	RV (*n* = 25,274)	NV (*n* = 441)	ADV (*n* = 1047)
**Respiratory diseases**				
Upper respiratory tract diseases	1 (1.20)	3641 (14.41)	86 (19.50)	211 (20.15)
Bronchitis	3 (3.61)	2991 (11.83)	48 (10.88)	100 (9.55)
Bronchopneumonia	0 (0)	1748 (6.92)	28 (6.35)	40 (3.82)
Pneumonia	2 (2.41)	1102 (4.36)	17 (3.85)	29 (2.77)
Asthma	0 (0)	26 (0.10)	0 (0)	0 (0)
Respiratory failure	0 (0)	28 (0.11)	0 (0)	3 (0.29)
Pulmonary vascular disease^a^	0 (0)	24 (0.09)	0 (0)	3 (0.29)
**Urogenital diseases**				
Hematuria and/or Proteinuria	0 (0)	5 (0.02)	0 (0)	0 (0)
Nephritis	0 (0)	5 (0.02)	0 (0)	0 (0)
Renal failure	0 (0)	93 (0.37)	0 (0)	1 (0.10)
Renal insufficiency	0 (0)	162 (0.64)	2 (0.45)	3 (0.29)
Nephrotic syndrome	0 (0)	12 (0.05)	0 (0)	0 (0)
Urethral diseases^a^	0 (0)	14 (0.06)	0 (0)	0 (0)
Urethral stone, obstruction and hydroureter	0 (0)	44 (0.17)	0 (0)	5 (0.48)
**Endocrine system diseases**				
Abnormal glucose metabolism	0 (0)	291 (1.15)	11 (2.49)	26 (2.48)
Diabetes	0 (0)	10 (0.04)	0 (0)	0 (0)
Obesity	0 (0)	1 (0.004)	0 (0)	0 (0)
Hyperinsulinemia	0 (0)	1 (0.004)	0 (0)	0 (0)
Pancreatitis	0 (0)	5 (0.02)	0 (0)	0 (0)
Thyroid function abnormalities	0 (0)	206 (0.82)	9 (2.04)	2 (0.19)
Rickets	0 (0)	9 (0.04)	0 (0)	0 (0)
**Immune system diseases**				
Kawasaki disease	0 (0)	22 (0.09)	2 (0.45)	1 (0.10)
Hypogammaglobulinemia	0 (0)	21 (0.08)	0 (0)	0 (0)
Antibody deficiency disorders	0 (0)	1 (0.004)	0 (0)	0 (0)
Immunoglobulin deficiency syndromes	0 (0)	49 (0.19)	1 (0.23)	0 (0)
** Skin and subcutaneous tissue diseases**^a^	2 (2.41)	739 (2.92)	13 (2.95)	25 (2.39)
**Nervous system diseases**				
Epilepsy	0 (0)	327 (1.29)	5 (1.13)	12 (1.15)
Seizure^b^	2 (2.41)	1415 (5.60)	64 (14.51)	63 (6.02)
* Febrile seizures*	*0 (0)*	*14 (0.99)*	*2 (6.25)*	*0 (0)*
* Benign convulsions with gastroenteritis*	*0 (0)*	*31 (2.19)*	*7 (21.88)*	*3 (7.14)*
* Benign infantile convulsions*	*0 (0)*	*218 (15.41)*	*23 (71.88)*	*18 (42.86)*
** Congenital disease**^a^	4 (4.82)	426 (1.69)	9 (2.04)	8 (0.76)
**Digestive system diseases**				
Digestive tract stenosis and obstruction	0 (0)	444 (1.76)	8 (1.81)	13 (1.24)
Gastrointestinal hemorrhage	0 (0)	140 (0.55)	1 (0.23)	5 (0.48)
Biliary tract or bile duct diseases	0 (0)	25 (0.10)	1 (0.23)	4 (0.38)
cholestasis	0 (0)	19 (0.08)	1 (0.23)	4 (0.38)
Hepatic failure	0 (0)	16 (0.06)	0 (0)	1 (0.10)
Hepatic dysfunction	5 (6.02)	1556 (6.16)	17 (3.85)	25 (2.39)
Other liver-related diseases^a^	0 (0)	240 (0.95)	6 (1.36)	10 (0.96)
Peptic ulcer	0 (0)	51 (0.20)	1 (0.23)	0 (0)
** Neonatal diseases**^a^	1 (1.20)	578 (2.29)	9 (2.04)	24 (2.29)
**Hematologic diseases**				
Leukocyte-related disorders	0 (0)	141 (0.56)	4 (0.91)	2 (0.19)
Neutrophilic diseases	4 (4.82)	1220 (4.83)	23 (5.22)	32 (3.06)
Platelet disorders	0 (0)	104 (0.41)	4 (0.91)	6 (0.57)
Hemolytic anemia	0 (0)	1 (0.004)	1 (0.23)	0 (0)
Nutritional anemia	1 (1.20)	448 (1.77)	5 (1.13)	36 (3.44)
Aplastic anemia	0 (0)	3 (0.01)	1 (0.23)	1 (0.10)
Thalassemia	0 (0)	19 (0.08)	0 (0)	0 (0)
Coagulation disorders	0 (0)	34 (0.13)	2 (0.45)	3 (0.29)
Favism	0 (0)	20 (0.08)	1 (0.23)	0 (0)
Langerhans cell histiocytosis	0 (0)	3 (0.01)	0 (0)	0 (0)
Hemophagocytic syndrome	0 (0)	4 (0.02)	1 (0.23)	0 (0)
Lymphadenoma	0 (0)	5 (0.02)	0 (0)	0 (0)
Leukemia	0 (0)	12 (0.05)	0 (0)	0 (0)
**Circulatory system diseases**				
Congenital heart disease	0 (0)	1186 (4.69)	13 (2.95)	65 (6.21)
Cardiac insufficiency	0 (0)	13 (0.05)	1 (0.23)	1 (0.10)
Myocardial diseases^b^	23 (27.71)	7845 (31.04)	75 (17.01)	115 (10.98)
* Abnormal cardiac enzymes*	*0 (0)*	*261 (3.33)*	*2 (2.67)*	*0 (0)*
* Myocardial impairment*	*23 (100)*	*6188 (78.88)*	*72 (96.00)*	*84 (73.04)*
* Myocarditis*	*0 (0)*	*22 (0.28)*	*0 (0)*	*2 (1.74)*
* Cardiomyopathy*	*0 (0)*	*11(0.14)*	*0 (0)*	*1 (0.87)*
Heart failure	0 (0)	8 (0.03)	0 (0)	2 (0.19)
Arrhythmia	2 (2.41)	95 (0.38)	0 (0)	3 (0.29)
Valvar heart diseases	0 (0)	20 (0.08)	0 (0)	0 (0)
Primary structural cardiac anomalies	0 (0)	7 (0.03)	0 (0)	1 (0.10)
Cardio vascular diseases	0 (0)	19 (0.08)	1 (0.23)	0 (0)
** Hereditary diseases**	0 (0)	31 (0.12)	2 (0.45)	0 (0)
**Nutritional diseases**				
Growth retardation	1 (1.20)	114 (0.45)	3 (0.68)	4 (0.38)
Vitamin deficiency	0 (0)	102 (0.40)	5 (1.13)	3 (0.29)
Malnutrition	7 (8.43)	522 (2.07)	16 (3.63)	29 (2.77)

*CV* Coxsackievirus, *RV* Rotavirus, *NV* Norovirus, *ADV* Adenovirus

^a^Pulmonary vascular disease: pulmonary artery stenosis, pulmonary valve stenosis, pulmonary artery fistula, etc. Urethral diseases: hypospadias, urethritis, urethral stricture, etc. Skin and subcutaneous tissue diseases: polymorphic rash, lymphadenopathy, etc. Congenital disease: trisomy 21 syndrome, congenital malformation, imperforate anus, congenital rubella syndrome, congenital hyperinsulinism, etc. other liver-related diseases: glycogen storage disease, fatty liver, hepatoblastoma, cirrhosis, etc. Neonatal diseases: neonatal septicemia, neonatal hypothermia, Neonatal hyperbilirubinemia, neonatal encephalopathy, etc.

^b^Numbers in italics represent subcategories in Seizure and Myocardial diseases. A definition for each of the subcategories of seizures and cardiomyopathy was shown in Supplementary Material 2

### Coinfection with other pathogens

 For 26,845 viral gastroenteritis cases with RV, CV, NV or ADV, the co-infection rate was 6.28% (1686/26845): viruses in 792 (2.95%), bacteria in 176 (0.66%), fungi and atypical pathogens in 718 (2.67%). The most commonly co-infection pathogens were *Mycoplasma pneumoniae* (*M. pneumoniae*) (1.95%, 523 cases), Epstein-Barr virus (EBV) (0.98%, 262 cases), influenza virus (FLU) (0.67%, 181 cases), cytomegalovirus (CMV) (0.53%, 141 cases), *candida albicans* (*C. albicans*) (0.48%, 129 cases), and respiratory syncytial virus (RSV) (0.41%, 110 cases) (Table 4). *M. pneumoniae* was detected more commonly in viral gastroenteritis children infected with RV or NV than with CV or ADV (2.01–2.27% vs. 0–0.57%). EBV (1.00%, 253/25274) and RSV (0.42%, 107/25274) were more commonly co-infected with RV in children with viral gastroenteritis, while CMV more commonly with CV (1.20%, 1/83) and FLU with NV (0.91%, 4/441) (Table 4).

**Table 4:** Positive number (n) and rate (%) of children hospitalized for viral gastroenteritis with other pathogens

Pathogens	CV (*n* = 83)	RV (*n* = 25,274)	NV (*n* = 441)	ADV (*n* = 1047)	Total (*n* = 26,845)
**Virus**					
EBV	0 (0)	253 (1.00)	4 (0.91)	5 (0.48)	262 (0.98)
HIV	0 (0)	2 (0.01)	0 (0)	0 (0)	2 (0.01)
HSV	0 (0)	29 (0.11)	0 (0)	1 (0.10)	30 (0.11)
RuV	0 (0)	1 (0.004)	0 (0)	0 (0)	1 (0.004)
PIV	0 (0)	41 (0.16)	4 (0.91)	0 (0)	45 (0.17)
HV	0 (0)	6 (0.02)	0 (0)	0 (0)	6 (0.02)
CMV	1 (1.20)	135 (0.53)	3 (0.68)	2 (0.19)	141 (0.53)
FLU	0 (0)	176 (0.70)	4 (0.91)	1 (0.10)	181 (0.67)
MV	0 (0)	3 (0.01)	0 (0)	0 (0)	3 (0.01)
VZV	0 (0)	6 (0.02)	0 (0)	0 (0)	6 (0.02)
RhV	0 (0)	2 (0.01)	1 (0.23)	0 (0)	3 (0.01)
RSV	0 (0)	107 (0.42)	1 (0.23)	2 (0.19)	110 (0)
SARS-CoV-2	0 (0)	2 (0.01)	0 (0)	0 (0)	2 (0.01)
**Bacteria**					
* A. baumannii*	0 (0)	1 (0.004)	0 (0)	0 (0)	1 (0.004)
* E. coli*	0 (0)	3 (0.01)	0 (0)	0 (0)	3 (0.01)
* S. flexneri*	0 (0)	1 (0.004)	0 (0)	0 (0)	1 (0.004)
* S. aureus*	0 (0)	2 (0.01)	0 (0)	0 (0)	2 (0.01)
* M. tuberculosis*	0 (0)	4 (0.02)	0 (0)	0 (0)	4 (0.01)
* Legionella*	0 (0)	26 (0.10)	0 (0)	1 (0.10)	27 (0.10)
* S. pneumoniae*	0 (0)	15 (0.06)	1 (0.23)	0 (0)	16 (0.06)
* S. aureus*	0 (0)	9 (0.04)	0 (0)	1 (0.10)	10 (0.04)
* Salmonella*^*a*^	0 (0)	38 (0.15)	2 (0.45)	6 (0.57)	46 (0.17)
* H. influenzae*	0 (0)	15 (0.06)	0 (0)	0 (0)	15 (0.06)
* B. pertussis*	0 (0)	6 (0.02)	0 (0)	1 (0.10)	7 (0.03)
* H. pylori*	0 (0)	11 (0.04)	0 (0)	0 (0)	11 (0.04)
Unknown bacteria	0 (0)	32 (0.13)	1 (0.23)	0 (0)	33 (0.12)
**Fungi and other pathogens**					
* C. albicans*	0 (0)	119 (0.47)	8 (1.81)	2 (0.19)	129 (0.48)
* Unknown fungi*	0 (0)	5 (0.02)	0 (0)	1 (0.10)	6 (0.02)
* A. lumbricoides*	0 (0)	7 (0.03)	0 (0)	1 (0.10)	8 (0.03)
* M. pneumoniae*	0 (0)	507 (2.01)	10 (2.27)	6 (0.57)	523 (1.95)
* C. pneumoniae*	0 (0)	51 (0.20)	0 (0)	0 (0)	51 (0.19)
* TP*	0 (0)	1 (0.004)	0 (0)	0 (0)	1 (0.004)

*CV* Coxsackievirus, *RV* Rotavirus, *NV* Norovirus, *ADV* Adenovirus, *EBV* Epstein-Barr virus, *HIV* Human Immunodeficiency Virus, *HSV* Herpes simplex virus, *RuV* Rubella virus, *PIV* Parainfluenza virus, *HV* Hepatitis virus, *CMV* Cytomegalovirus, *FLU* Influenza virus, *MV* Measles virus, *VZV* Varicella-zoster virus, *RhV* Rhinovirus, *RSV* Respiratory syncytial virus, *SARS-CoV-2* Severe acute respiratory syndrome coronavirus 2, *A. baumannii*: *Acinetobacter baumannii*, *E.coli*: *Escherichia coli*, *S. flexneri*: *Shigella flexneri*, *S. aureus*: *Staphylococcus aureus*, *M. tuberculosis*: *Mycobacterium tuberculosis*, *S. pneumoniae*: *Streptococcus pneumoniae*, *S. aureus*: *Staphylococcus aureus*, *H. influenzae*: *Haemophilus influenzae*, *B. pertussis*: *Bordetella pertussis*, *H. pylori*: *Helicobacter pylori*, *C. albicans*: *Candida albicans*, *A. lumbricoides*: *Ascaris lumbricoides*, *M. pneumoniae*: *Mycoplasma pneumoniae*, *C. pneumoniae*: *Chlamydia pneumoniae*, *TP*: *Treponema pallidum*

^a^The *Salmonella bacteria* include *Salmonella typhimurium* (*S. typhimurium*), *Salmonella Choleraesuis* (*S. Choleraesuis*) and other unclassified strains

### Complications

Complications of viral gastroenteritis included dehydration, acidosis, electrolyte disorders and shock (Table 5). Children aged 28 days <  ~  ≤ 1 year were most likely to suffer from acidosis, while those aged 6 <  ~  ≤ 12 years old were most likely to suffer from dehydration (*P* < *0.001*). Dehydration was more common in children with NV infection, while electrolyte disturbances were more prevalent in children with RV infection, and acidosis with ADV infection (*P* < *0.001*).

**Table 5:** Positive number (n) and rate (%) of children hospitalized for viral gastroenteritis with complications from Jan 1st, 2016 to Dec 31st, 2020

Characteristic	Dehydration (n, %)	Electrolyte disorders (n, %)	Acidosis (n, %)	Shock (n, %)	Total (n, %)
Age group					
≤ 28 days	101(13.78)^a^	22(3.00)^a^	113 (15.42)^a^	2(0.27)	733
28 days < ~ ≤ 1 years old	7491(42.65)^b^	1944(11.07)^b^	5726(32.60)^b^	34(0.19)	17,563
1 < ~ ≤ 3 years old	3481 (39.54)^c^	1191(13.53)^c^	2388(27.13)^c^	15(0.17)	8803
3 < ~ ≤ 6 years old	326(33.99)^d^	119(12.41)^b,c^	226(23.57)^d^	1 (0.10)	959
6 < ~ ≤ 12 years old	26(20.97)^e^	20(16.13)^b,c^	13(10.48)^a^	0(0.00)	124
> 12 years old	1(14.29)^a,b,c,d,e^	0(0.00)^a,b,c^	2(28.57)^a,b,c,d^	0(0.00)	7
χ^2^	292.674	92.778	206.708	1.062	
* P value*	< 0.001	< 0.001	< 0.001	0.975	
Etiology					
CV	11(13.25)	6(7.23)^a,b^	3 (3.61)	1(1.20)	83
RV	10,455(41.37)	3040(12.03)^b^	7670(30.35)^a^	43(0.17)	25,274
NV	226 (51.25)	45(10.20)^b^	134(30.39)^a,b^	1 (0.23)	441
ADV	327(31.23)^a^	112(10.70)^b^	359(34.29)^b^	4(0.38)	1047
Not clear	407(30.28)^a^	93(6.92)^a^	302 (22.47)	3(0.22)	1344
χ^2^	150.075	35.962	74.377	7.347	
* P value*	< 0.001	< 0.001	< 0.001	0.119	

Letters “a” “b” “c” “d” and “e” showed that there was different between groups in the different groups

*CV* Coxsackievirus, *RV* Rotavirus, *NV* Norovirus, *ADV* Adenovirus

### LOS, hospitalization expense, discharge and outcome

The median length of stay (LOS) for hospitalized viral gastroenteritis patients was 5 days (IQR: 3–6 days), and the median expense was 587 USD (541–642 USD) (Table 6). The LOS was shortest for children aged 1 <  ~  ≤ 3 years and longest for children aged 28 days <  ~  ≤ 1 year. Total costs were highest among hospitalized patients aged ≤ 28 days(*P* < *0.001*). Children with RV gastroenteritis had the longest LOS (*P* < *0.001*). The hospital cost was the highest with ADV gastroenteritis (642 US$) and lowest with RV gastroenteritis (541 US$). The majority of hospitalized patients recovered and were able to be discharged within a few days, in which the percentage of discharge by patient with medical advice was 93.5% (26,363 cases) (Table 1). Fortunately, there were no deaths in our study.

**Table 6:** LOS and hospitalization expense of children with viral gastroenteritis (Median and IQR)

Characteristic	Length of stay (days)	Cost (US dollars)	χ^2^	*P value*
Total	5(3–6)	587(541–642)		
Age group			242.05/505.92	< 0.0001
≤ 28 days	5(4–7)^a^	758(555–1035)^a^		
28 days < ~ ≤ 1 years old	5(4–6)^a,b^	561(390–778)^b^		
1 < ~ ≤ 3 years old	5(4–6)^c^	515(371–703)^c^		
3 < ~ ≤ 6 years old	4(3–6)^d^	515(353–695)^c^		
6 < ~ ≤ 12 years old	4(3–6)^c,d^	548(365–740)^b,c^		
> 12 years old	6(5–8)^a,b,c,d^	810(366–1226)^a,b,c^		
Regions			688.09/2,436.03	< 0.0001
Northeast	5(4–6)^a^	566(448–733)^a^		
North China	5(4–6)^a^	643(477–876)		
East China	5(4–6)	473(347–641)		
South China	4(3–5)	379(279–536)		
Central China	5(4–7)^a^	442(324–632)		
Northwest	5(3–6)	659(450–844)		
Southwest	5(4–6)	562(426–736)^a^		
Etiology			76.76/170.92	< 0.0001
CV	5(3–6)^a,b,c,d,e^	541(435–691)^a,b,c,d,e^		
RV	5(4–6)^b,d^	541(379–751)^b^		
NV	5(4–6)^c^	627(468–835)^a,c,d,e^		
ADV	5(3–6)^d^	642(453–835)^a,c,d,e^		
Not clear	4(3–6)^e^	586(435–811)^a,c,d,e^		

Letters “a” “b” “c” “d” and “e” showed that there was different between groups in the different groups

*CV* Coxsackievirus, *RV* Rotavirus, *NV* Norovirus, *ADV* Adenovirus

## Discussion

This study summarized and analyzed the FSMRs data of 28,189 hospitalized children with viral gastroenteritis from 2016 to 2020 in the mainland of China to provide further evidence for the role of gastrointestinal viral infections in this most common gastrointestinal emergency in children. We found that the burden of viral gastroenteritis related hospitalization was the highest among children younger than 3 years of age. RV, CV, ADV and NV accounted for 95.2% of the children with viral gastroenteritis. We demonstrated that the coexistence rates of viral gastroenteritis with upper respiratory tract diseases, myocardial diseases, or seizure were high, and the co-occurrence of other viral, bacterial or atypical pathogen infections was common in pediatric patients hospitalized with viral gastroenteritis, such as *M. pneumoniae*, EBV and CMV.

Children with viral gastroenteritis under 18 years of age have a ratio of 1.54 to1 between males and females. The result coincided with previous research in China, which showed the ratio was 1.68:1 for children under 5 years of age with gastroenteritis in western China from 2015 to 2019 [14]. Our data suggests that children bearing the greatest burden of hospitalization associated with AGE especially infected with RV were children younger than 3 years (96.3%). In comparison with other studies worldwide [15–17], there is a significant difference in the detection rate of rotavirus and norovirus. This may be attributed to the involvement of multiple hospitals in this study, each of which adopted different diagnostic methods. Additionally, RV infection often results in fever, vomiting, dehydration, and severe diarrhea compared to NV infection, increasing the likelihood of hospitalization [18–20]. This study specifically focused on hospitalized patients with viral gastroenteritis, rather than the entire population or patients with diarrhea. It’s important to consider that hospitalized patients usually have more severe conditions than outpatients, which may explain the significantly higher detection rate of RV compared to NV. This study clearly showed the seasonal characteristics of the hospitalized cases in children with viral gastroenteritis over the past five years. The fluctuation of the total number and rate were mainly caused by the change in RV, and the infection of rotavirus mainly occurs from November to March, which is in accordance with previous studies from other countries and other regions of China [9, 21–23].

In China, the Lanzhou lamb rotavirus (LLR) vaccine was licensed and has been available since 2000 in China, RotaTeq (RV5) in 2018, and Lanzhou lamb reassortant rotavirus vaccine, live, oral, trivalent (Vero cell) (LLR3) in 2023. Even they have not been included in NIPs, the number of rotavirus vaccine doses produced and administered in China has been increasing [24, 25]. RV showed the smallest number and lowest proportion in 2020, which can demonstrate that the contribution of RV to pediatric acute gastroenteritis will gradually decrease as the application of vaccine. Moreover, the COVID-19 (coronavirus disease 2019) pandemic may have altered the epidemiological landscape of various pathogens. In response to COVID-19, control strategies such as social distancing, lockdowns, and enhanced personal hygiene standards were implemented, affecting the transmission of pathogens [26–29]. A retrospective study on Chinese Taiwan children revealed that RV hospitalization rates among children < 5 years of age significantly declined by 24.0% in post-vaccine compared to pre-vaccine rotavirus seasons [30]. A multicenter study in China from 2003 to 2012 showed a 70% decrease in the mortality rate of rotavirus gastroenteritis (RVGE) in 2012 compared to 2003 [2]. So, the increasing use of rotavirus vaccines can diminish the burden and change the epidemiology of rotavirus disease worldwide especially in developing countries, as Shim et al. showed that vaccines had a protective effect for hospitalized children with acute gastroenteritis [31]. Advancements in the medical infectious disease reporting system, pathogen detection technology, and awareness about sending specimens for testing for pathogens have resulted in the detection of more positive cases. The first-dose RV vaccine coverage in China was 20.3%, with only 1.8% coverage for the third dose; consequently, despite a decrease in hospitalization and mortality rates for RVGE in China, the disease burden persists [32].

Viral gastroenteritis combined with myocardial diseases refers to the condition where viral infection leads to myocardial damage and myocarditis [33, 34]. In this study, the proportion of viral gastroenteritis combined with myocarditis was high, ranging from 10.98% to 31.04%, in which over 70% of cases were diagnosed with myocardial injuries. Cioc et al. revealed that among 13 cases of sudden cardiac arrest patients, 5 cases of CV and 4 cases of RV in myocardial tissue samples, and a number of case reports have described especially myocarditis of RV infection [35, 36]. Viral gastroenteritis can also be associated with central nervous system (CNS) diseases, known as encephalitis or meningitis. This study found that the proportion of cases with seizures was from 2.41–14.51%, in which benign infantile convulsions had the highest proportion, and benign convulsions with gastroenteritis only accounted for 0–21.88% of cases with seizures, similar to other reports [37–39]. There have been numerous cases with respiratory diseases, including 60 cases (6.59%) of ADV-associated pneumonia and bronchopneumonia. According to previous studies, ADV is more commonly cause gastrointestinal diseases by serotypes 40 or 41 [40, 41]. Therefore, for children hospitalized with viral gastroenteritis, we should pay attention to the possibility of concurrent other systemic diseases and remain vigilant for the occurrence of severe cases.

In terms of the co-infection with other pathogens, *M. pneumoniae*, EBV, FLU, and CMV were the main pathogens of viral gastroenteritis in hospitalized children. *M. pneumoniae* and FLU infections usually cause respiratory diseases, such as *Mycoplasma pneumoniae* pneumonia and viral pneumonia, which aligned with the high proportion of respiratory diseases among children with viral gastroenteritis that were mentioned earlier. In this study EBV-positive cases included infectious mononucleosis, EBV viremia, and other EBV-related diseases. It is important to note that a positive result does not necessarily indicate that EBV infection will result in disease or symptoms, because most patients may have latent EBV infection [42]. Similar to EBV, CMV is typically asymptomatic in the majority of individuals. However, in children with compromised immune function who are hospitalized with viral gastroenteritis, CMV infection can cause clinical symptoms and complications, such as hepatitis, pneumonia, and impairment of the brain and visual system. For children with viral gastroenteritis, properly controlling infections caused by other pathogens is crucial to immunocompromised patients, including immune deficiencies, hematopoietic stem cell transplantation or liver transplantation [43, 44].

AGE is generally a self-limiting condition and resolves within one week, which is most commonly associated with viral infection [45–47]. There were no fatal cases in our study, as a result of viral gastroenteritis, patients usually suffered from complications, such as dehydration, electrolyte disorders, and acidosis. Our study showed there was more dehydration in children with NV infections, electrolyte disturbances in children with RV infections, and electrolyte disorders with ADV infections. LOS and hospitalization expense of children with viral gastroenteritis were associated with age, region and pathogens in children.

Our study has some limitations. The most frequently seen patients with viral gastroenteritis are outpatients, while our database only contains the data generated from hospitalized children, so little is known regarding the total incidence of viral gastroenteritis. Meanwhile, various detection methods for pathogens in different hospitals lead to the effectiveness of the detection method for the same pathogen is diverse, so we are unable to make comparisons between different pathogens. In this study, no follow-up information was available for the children with viral gastroenteritis, especially patients who had been transferred to other hospitals. Due to a lack of clear information regarding the patient's RV vaccination status, it is hard to assess the protective effect of RV vaccination in viral gastroenteritis.

## Conclusion

Rotavirus was detected in nearly 90% of children with viral gastroenteritis among hospitalized children in the mainland of China. Most cases occurred among children younger 3 years during the winter months. Children with viral gastroenteritis were often accompanied by other diseases and pathogens, with myocardial diseases the most common disease and *M. pneumoniae* the most common pathogen. Continuous surveillance is needed to monitor the prevalence of viral gastroenteritis, and the immunization schedule of rotavirus is essential for adequate management of viral gastroenteritis.

### Supplementary Information

**Supplementary Material 1.**

**Supplementary Material 2.**

## Data Availability

The dataset used and/or analyzed during the current study are available from the corresponding author on reasonable request.

## Abbreviations

AGE: Acute gastroenteritis
FRCPD: Futang Children's Medical Development Research Center
RV: Rotavirus
NV: Norovirus
ADV: Adenovirus
CV: Coxsackievirus
EV: Enterovirus
*M. pneumoniae*: *Mycoplasma pneumoniae*
EBV: Epstein-Barr virus
FLU: Influenza virus
AV: Astrovirus
NIPs: National Immunization Programs
FSMRs: Face sheet of discharge medical records
ICD-10: International Statistical Classification of Diseases and Related Health Problems 10th Revision
IQR: Interquartile range
CMV: Cytomegalovirus
*C. albicans*: *Candida* *albicans*
RSV: Respiratory syncytial virus
LOS: Length of stay
WHO: World Health Organization
LLR: Lanzhou lamb rotavirus
RV5: RotaTeq
LLR3: Lanzhou lamb reassortant rotavirus vaccine, live, oral, trivalent (Vero cell)
RVGE: Rotavirus gastroenteritis
CNS: Central nervous system
