# Supplementary material for: Changes in the epidemiology and clinical characteristics of viral gastroenteritis among hospitalized children in the Mainland of China: a retrospective study from 2016 to 2020
Source: BMC Pediatr. 2024 May 4;24:303. doi: 10.1186/s12887-024-04776-1 (PMC11069194; doi:10.1186/s12887-024-04776-1)
Supplement: Supplementary file 1 — Supplementary Material 1. [file 12887_2024_4776_MOESM1_ESM.docx]

| Regions | Hospital in FUTURE database |
| --- | --- |
| Northeast China (3) |  |
|  | Changchun Children’s Hospital |
|  | Liaoning Children’s Hospital |
|  | Dalian Women and Children’s Medical Group |
| North China (5) |  |
|  | Beijing Children’s Hospital, Capital Medical University |
|  | Baoding Children’s Hospital |
|  | Children’s Hospital of Hebei Province |
|  | Children’s Hospital of Shanxi |
|  | Inner Mongolia Maternity and Child Health Care Hospital |
| East China (8) |  |
|  | Children’s Hospital Affiliated to Shandong University |
|  | Women and Children Hospital in Liaocheng |
|  | Children’s Hospital of Nanjing Medical University |
|  | Children’s Hospital of Soochow University |
|  | Hangzhou Children’s Hospital |
|  | Fuzhou Children’s Hospital of Fujian Province |
|  | Anhui Province Children’s Hospital |
|  | Jiangxi Province Children’s Hospital |
| South China (2) |  |
|  | Liuzhou Maternity and Child Healthcare Hospital |
|  | Shenzhen Children’s Hospital |
| Central China (3) |  |
|  | Henan Children’s Hospital |
|  | Hunan Children’s Hospital |
|  | Wuhan Children’s Hospital, Tongji Medical College, Huazhong University of Science & Technology |
| Northwest China (4) |  |
|  | Gansu Provincial Maternity and Child-care Hospital |
|  | Urumqi Children’s Hospital |
|  | Qinghai Province Women and Children’s Hospital |
|  | Xi’an Children’s Hospital |
| Southwest China (2) |  |
|  | Guiyang Children’s Hospital |
|  | Kunming Children’s Hospital |

**Table S1** The geographic distribution of 27 children’s hospital in the FUTang Updating medical REcords (FUTURE) database
