# Supplementary material for: Changes in the epidemiology and clinical characteristics of viral gastroenteritis among hospitalized children in the Mainland of China: a retrospective study from 2016 to 2020
Source: BMC Pediatr. 2024 May 4;24:303. doi: 10.1186/s12887-024-04776-1 (PMC11069194; doi:10.1186/s12887-024-04776-1)
Supplement: Supplementary file 2 — Supplementary Material 2. [file 12887_2024_4776_MOESM2_ESM.docx]

**Supplementary Material 2.** The definition for each of the subcategories of seizures and cardiomyopathy

1. A seizure is a burst of uncontrolled electrical activity between brain cells (also called neurons or nerve cells) that causes temporary abnormalities in muscle tone or movements (stiffness, twitching or limpness), behaviors, sensations or states of awareness.

1. Febrile seizure (FS) is the most common type of childhood seizure disorder, which occurs in an age-specific manner, is associated with a fever of 38.0℃ or higher, and presents without evidence of any definite causative diseases, such as central nervous system (CNS) infection or metabolic abnormality. (PMID: 25324864)
2. Benign convulsions with mild gastroenteritis (CwG) are characterized by afebrile convulsions associated with viral gastroenteritis in previously healthy infants and children. The main causative pathogens are rotavirus and norovirus. (PMID: 25114690)
3. Benign familial infantile seizure is a genetic epilepsy syndrome with autosomal dominant inheritance. It may be associated with paroxysmal choreoathetosis (infantile convulsions and choreoathetosis syndrome), which has been linked to the chromosome 16 infantile convulsions and choreoathetosis syndrome region. (PMID: 12503648)

2. Myocardial diseases include different diseases caused by multiple causes, some of which are still unknown. Myocardial diseases mainly include myocarditis (such as viral myocarditis), cardiomyopathy, and cardiac tumors. (the eighth edition of Zhu Futang Practical Pediatrics)

1. Elevated levels of cardiac enzymes (cardiac biomarkers) in the blood are a sign of heart damage, stress or inflammation.
2. Myocardial impairment refers to pathological damage to the myocardium caused by various pathological factors, presenting clinical symptoms such as chest tightness, shortness of breath, and palpitations, which may be accompanied by an increase in myocardial enzymes. However, clinically it does not meet the diagnostic criteria for myocarditis, and it is a temporary diagnosis.
3. Myocarditis is a serious though rare condition where inflammation develops in the myocardium, or middle muscular layer of the heart wall.
4. Any disorder that affects the heart muscle is called a cardiomyopathy.
